# Drought-Induced Xylem Sulfate Activates the ABA-Mediated Regulation of Sulfate Assimilation and Glutathione Redox in *Brassica napus* Leaves

**DOI:** 10.3390/metabo12121190

**Published:** 2022-11-29

**Authors:** Bok-Rye Lee, Sang-Hyun Park, Van Hien La, Dong-Won Bae, Tae-Hwan Kim

**Affiliations:** 1 Grassland Science Laboratory, Department of Animal Science, Institute of Agricultural Science and Technology, College of Agriculture & Life Science, Chonnam National University, Gwangju 61186, Republic of Korea; 2 Institute of Environmentally-Friendly Agriculture (IEFA), Chonnam National University, Gwangju 61186, Republic of Korea; 3 Center of Crop Research for Adaption to Climate Change (CRCC), Thai Nguyen University of Agriculture and Forestry, Thai Nguyen 24000, Vietnam; 4 Biomaterial Analytical Laboratory, Central Instruments Facility, Gyeongsang National University, Jinju 52828, Republic of Korea

**Keywords:** abscisic acid, *Brassica napus*, drought, glutathione, redox, stress intensity, sulfate

## Abstract

Drought intensity modifies the assimilatory pathway of glutathione (GSH) synthesis. Abscisic acid (ABA) is a representative signaling hormone involved in regulating plant stress responses. This study aimed to investigate an interactive regulation of sulfate and/or ABA in GSH metabolism and redox. The drought-responsive alterations in sulfate assimilation and GSH-based redox reactions were assessed relative to ABA responses on the time-course of drought intensity. Drought-responsive H_2_O_2_ concentrations were divided into two distinct phases—an initial 4 days of no change (*Ψ_w_* ≥ −0.49 MPa) and a phase of higher accumulation during the late phase of the drought (days 10–14; *Ψ_w_* ≤ −1.34 MPa). During the early phase of the drought, GSH/GSSG redox state turned to the slightly reduced state with a transient increase in GSH, resulting from a strong activation of H_2_O_2_ scavenging enzymes, ascorbate peroxidase (APOX) and glutathione reductase (GR). The late phase of the drought was characterized by a decrease in GSH due to cysteine accumulation, shifting GSH- and NADPH-based redox states to higher oxidization, increasing sulfate and ABA in xylem, and causing ABA accumulation in leaves. Regression analysis revealed that sulfate in xylem sap was positively correlated with H_2_O_2_ concentrations and ABA was closely related to decreases in the GSH pool and the oxidation of GSH catalyzed by glutathione peroxidase (GPOX). These results indicate that drought-induced oxidation proceeds through the suppression of GSH synthesis and further GSH oxidation in a sulfate-activated ABA-dependent manner.

## 1. Introduction

Drought is the most common adverse environmental stress that limits plant growth and development in worldwide. In Korea, oilseed rape (*Brassica napus* L.) is often exposed to drought stress during the early stages of its growth since drought occurs predominantly from March to early June, which is the active growth and development period of most winter plant varieties. Decreasing water availability during droughts generally results in reduced total nutrient uptake, leading to reduced concentrations of mineral nutrients in plants. Sulfur (S) deficiency in plant cells reduces chlorophyll and Rubisco content and promotes chlorosis in young leaves [1,2,3], resulting in a general inhibition of photosynthesis and protein synthesis [4]. Thus, S-use efficiency has proven to be a significant determinant of drought-stress tolerance, specifically regarding photosynthetic activity in oilseed rape cultivars [4]. The closure of stomata triggered by drought results in a rapid decline of the plant’s internal CO_2_/O_2_ ratio, inducing a high flux of reactive oxygen species (ROS) production in the peroxisomes through photorespiration [5,6]. Among ROS, low steady-state levels of H_2_O_2_ function as signal transduction molecules [7,8]. However, excessive ROS becomes deleterious and results in oxidative damage to membranes (i.e., lipid peroxidation), proteins, RNA, and DNA in a process termed “oxidative stress” [5,9,10]. Subsequent studies have focused on disequilibrium in the cellular reduction–oxidation status, called “redox” status, as a cause of oxidative stress. S metabolism is an essential in the regulation of cellular redox homeostasis under stress; for example, thiol-containing compounds, especially reduced GSH (sensitive to oxidized environments), can be modulators of the stress response [11,12]. H_2_O_2_ is reduced to H_2_O by ascorbate, while reduced GSH is converted to glutathione disulfide (GSSG) upon oxidation with two other GSH molecules [13]. The resultant oxidized glutathione (GSSG) is recycled back to GSH by the action of glutathione reductase (GR), using NADPH as the reductant [14,15], thereby maintaining the redox potential of GSH. Although GSH, which depends on the availability of S assimilates from sulfate reduction pathway (especially cysteine), is an essential regulator in stress response and tolerance [11,16,17], the sulfate assimilation pathway towards GSH-based redox in response to drought-induced oxidative stress has rarely been investigated.

Several studies have provided evidence for significant co-regulation between S metabolism and abscisic acid (ABA) biosynthesis that highlights the importance of S for stress tolerance [18,19,20]. ABA is a well-characterized signaling molecule that induces stomatal closure [19,20] and stress symptomatic responses [6,8,21], along with stress-induced ROS accumulation. Sulfate has been shown to promote ABA synthesis [20] and sulfate–ABA interactions lead to a greater reduction in transpiration rates and stomatal aperture than ABA alone [18]. Studies have shown that sulfate is a xylem-born chemical signal as ABA synthesis and is transported earlier than ABA [18,22]. Exogenous sulfate- or cysteine (Cys) up-regulates the expression of the 9-sis-epoxycarotenoid dioxygenase (*NCED3*), which is a key step during ABA synthesis [20,22]. Furthermore, sulfate assimilation into Cys is required to trigger ABA biosynthesis and stomatal closure [19] and ABA-triggered stomatal closure is a result of Cys accumulation and is not influenced by lowered GSH [23,24]. Taken together, previous studies have provided evidence for an interactive biological function between sulfate and ABA involved in regulating stress responses and stress tolerance in vascular plants. However, little understanding has been developed in relation to how sulfate or S-assimilates regulate GSH-based redox in possible interactions with ABA as being linked to drought-induced H_2_O_2_ accumulation.

In the present study, we hypothesized that (1) the endogenous levels of sulfate assimilates and ABA in shoots, roots, as well as in xylem sap are greatly influenced by drought intensity-responsive endogenous H_2_O_2_ levels, and (2) the interconnected signaling and metabolic pathways between sulfate and ABA are involved in GSH-based redox control. To test these hypotheses, endogenous ABA, sulfate and S- assimilates, antioxidative enzymes activity, xylem-borne sulfate and ABA were assessed in relation to endogenous H_2_O_2_ levels in *Brassica napus* leaves, based on the time-course of drought intensity.

## 2. Materials and Methods

### 2.1. Plant Growth and Treatments

*Brassica napus* L. (cv. Capitol) seeds were sown in bed soil in a tray. When the plants reached the four-leaf stage, seedlings were transferred to 2 L pots containing a mixture of soil and perlite (70:30, *w*/*w*) inside a greenhouse. A complete nutrient solution was continuously supplied to the plants [25] and metal halide lamps (c. 400 μmol photons m^−2^ s^−1^ at the canopy for 6 h per day) were used to supply natural light. Plants were selected according to morphological similarities after 6 weeks and were divided into two groups, one that was irrigated with 200 mL of water for well-watered plants (control) and the other that was irrigated with 20 mL of water for drought-stressed plants. Sampling was performed consecutively at 0, 2, 4, 10 and 14 days after the drought treatment.

### 2.2. Measurement of Leaf Water Potential

For measurement of leaf water potential (*Ψ_w_*), the seventh leaf was cut and placed in the pressure chamber (PMS Instruments, Corvallis, OR, USA) to expose the cut end of the petiole to the outside. Subsequently, pressure was applied to the chamber until liquid was observed at the end of the petiole, which corresponds to the *Ψ_w_*.

### 2.3. Collection of Xylem and Phloem

Xylem samples were obtained by cutting stems. After thorough washing of the stem’s surface, the exuding fluid was collected under high pressure using an exhausted syringe. Phloem exudates were collected in accordance with the methods described by Lee et al. [10]. The petiole of the leaf rank number of 3 (i.e., leaf No. 1 was the oldest leaf) was cut and immersed rapidly in 20 mL of EDTA solution (pH 7.0) for 5 min. The leaf was rinsed, transferred to another 15 mL of 5 mM EDTA solution (pH 7.0), and maintained for 6 h in a growth chamber with 95% relative humidity in darkness. The resulting collection of fluid samples was stored at −80 °C for further analysis.

### 2.4. Measurement of H_2_O_2_ Concentration

To determine H_2_O_2_ concentrations, approximately 200 mg of well-ground fresh leaves were homogenized with 1.2 mL of 100 mM phosphate buffer (pH 7.0) and the mixture was then centrifuged at 10,000× *g* for 10 min. The resulting extract (0.2 mL) was mixed with 0.2 mL of 0.1% titanium chloride in 20% (*v*/*v*) H_2_SO_4_ and was then centrifuged at 6000× *g* for 3 min. Absorbance was immediately read at 410 nm and the H_2_O_2_ concentration calculated from the extinction coefficient was found to be 0.28 μM^−1^ cm^−1^ [26].

### 2.5. Determination of ABA Concentration

Quantitative analysis of ABA in leaves, roots and xylem was performed according to the protocol used by La et al. [27]. Briefly, 50 mg of fresh ground tissues and 50 μL of concentrated xylem were extracted with 500 μL of extraction solvent (2-propanol/H_2_O/concentrated HCl; 5:1:0.002, *v*/*v*/*v*) containing d6-ABA as the internal standard for ABA. Dichloromethane (1 mL) was added to the supernatant and then centrifuged at 13,000× *g* for 5 min at 4 °C. The lower phase, which was taken into a clean screw-cap glass vial, was dried under nitrogen and resolved in pure methanol. Complete dissolved extract was ensured by vortexing and sonicating was transferred to a reduced volume liquid chromatography vial. The level of ABA was measured using a reverse-phase C18 Gemini high-performance liquid chromatography (HPLC) column for HPLC electrospray ionization tandem mass spectrometry (HPLC–ESI–MS/MS) analysis. Agilent 1100 HPLC (Agilent Technologies, Waldbronn, Stuttgart, Germany), Waters C18 column (150 × 2.1 mm, 51 μm), and API4000 MSMRM (Applied Biosystems, Waltham, MA, USA) were used for analysis.

### 2.6. Sulfate and Cys Concentration

To measure the concentration of sulfate, approximately 100 mg of leaves and roots was homogenized with 1.5 mL of extraction buffer containing 1.8 mM Na_2_CO_3_ and 1.7 mM NaHCO_3_ (*v*/*v* = 1:1). Samples were centrifuged at 12,000× *g* for 10 min, after which the supernatant was filtered through a 0.2-μm membrane-type filter, as well as concentrated xylem. Sulfate concentrations were determined by using ion chromatography (Dionex, DX-120, Sunnyvale, CA, USA), using an isocratic Na_2_CO_3_/NaHCO_3_ eluent (1.8 mM/1.7 mM) with a flow rate of 2.3 mL min^−1^. To determine the Cys content within plant tissue, approximately 200 mg of freshly ground samples was extracted using 1 mL of Na-PO_4_ (pH 7.5). The aliquot was then isolated and added to an acid ninhydrin solution (i.e., 0.25 g of acid ninhydrin mixed with 6 mL of acetic acid and 4 mL of concentrated HCl), after which it was heated at 100 °C for 10 min and was then cooled in ice. The absorbance was determined at 560 nm and Cys content was calculated using a standard curve [28].

### 2.7. Measurement of GSH/GSSG

For total GSH extraction, 200 mg of fresh leaves were homogenized in a 5% solution of 5-sulfosalicylic acid and centrifuged at 12,000× *g* for 10 min. GSSG sample were prepared by adding a thiol scavenger (GT40c, Oxford Biomedical Research Inc., Rochester Hills, MI, USA) immediately to the total GSH-extracted solution. The total GSH and GSSG contents of the resulting supernatants were then determined through a microplate assay using the GSH/GSSG Kit GT40 (Oxford Biomedical Research Inc., Rochester Hills, MI, USA). The extracted solution for total GSH and GSSG were mixed with glutathione reductase, DTNB (5,5-dithiobisnitrogenzoic acid) and NADPH. The absorbance was immediately recorded at 405 nm by taking readings every minute for 10 min. Total GSH and GSSG concentration were calculated using standard curve of GSH and GSSG, respectively. GSH concentration was obtained by subtracting GSSG from total GSH.

### 2.8. Measurement of NADPH/NADP^+^

The contents of oxidized and reduced pyridine nucleotides were measured as described previously [27]. For NADP^+^ and NADPH extraction, 200 mg of fresh leaves were homogenized with 0.8 mL of 0.2 N HCl and 0.2 M NaOH, respectively; 100 mL of this extract was heated at 95 °C for 1 min and was then placed in an ice-bath. For the NADP^+^ assay, the supernatant was neutralized with 0.2 M NaOH to a final pH of 5–6, while NADPH was neutralized by 0.2 N HCl to a final pH of 7–8 for the NADPH assay. Forty microliters were added to the reaction mixture containing 0.1 M HEPES (pH 7.5), which consisted of 2 mM Na_2_EDTA, 1.2 mM dichlorophenolindophenol, 20 mM phenazine methosulfate, and 10 mM glucose-6-phosphate for NADPH/NADP^+^. The reaction was started by adding 2 μL of glucose 6-phosphate dehydrogenase (200 U) for NADPH/NADP^+^ and the content of NADP^+^ and NADPH were quantified using a standard curve.

### 2.9. Measurements of Antioxidant Enzyme Activities

Well-ground leaves were extracted with 100 mM of potassium phosphate buffer (pH 7.5) containing 2 mM of ethylenediaminetetraacetic acid, 1% polyvinylpyrrolidone, and 1 mM of phenylmethylsulfonyl fluoride, then centrifuged at 15,000× *g* for 20 min at 4 °C. Superoxide dismutase (SOD) activity was determined based on the capacity of the enzyme to inhibit the photoreduction of nitroblue tetrazolium (NBT) [27]. The supernatant was mixed with 63 μM NBT, 1.3 μM riboflavin, 13 mM methionine, 100 mM potassium phosphate buffer (pH 7.0). The reaction mixture kept at dark condition for 30 min and exposed to light condition for 20 min. One unit of SOD was defined as the amount of enzyme that inhibited the rate of NBT photoreduction by 50% at 560 nm. For measurement of ascorbate peroxidase (APOX) activity, 100 mM potassium phosphate buffer (pH 7.5), 0.2 mM H_2_O_2_, and 0.5 mM ascorbate were added to the enzyme extract. APOX activity was calculated by monitoring the decrease in absorbance at 290 nm for 1 min as AsA (coefficient, ε = 2.8 mM^−1^ cm^−1^) was oxidized [26]. Glutathione reductase (GR) activity was measured using the method used by Hasanuzzaman et al. [29]. The enzyme extract was reacted with 100 mM potassium phosphate buffer (pH 7.8), 1 mM EDTA, 0.2 mM NADPH, and 1 mM GSSG. The decrease in absorbance at 340 nm, as the NADPH (coefficient, ε = 6.2 mM^−1^ cm^−1^) was oxidized. For the measurement of glutathione peroxidase (GPOX) activity, 100 mM sodium phosphate buffer (pH 7.5), 2 mM GSH, 1 mM EDTA, 1 mM NaN_3_, 0.12 mM NADPH, 1 unit GR, and 0.6 mM H_2_O_2_ were added to the enzyme extract. The reaction was initiated with the addition of H_2_O_2_ and monitored the decrease in the NADPH absorbance at 340 nm for 1 min [29]. The activity was then calculated using an extinction coefficient of 6.62 mM^−1^ cm^−1^.

### 2.10. RNA Extraction and Quantitative Real-Time PCR Analysis

For total RNA isolation, fresh leaves (200 mg) were mixed with RNAiso Plus reagent (Takara, Nojihigashi 7-4-38 Kusatsu, Shiga, Japan), keep at room temperature for 5 min, and then centrifuged at 12,000× *g* for 5 min at 4 °C. The solution was separated into three layers, among them, the top liquid layer collected and mixed with isopropanol. After 10 min at room temperature, the mixed solution was centrifuged at 12,000× *g* for 10 min at 4 °C to precipitate the RNA. The pellet was washed with an equivalent amount of 75% ethanol, dried and dissolved with DEPC-treated water. Genomic DNA was digested with DNase I. First-strand complementary DNA (cDNA) was synthesized with a GoScript Reverse Transcription System (Takara). The qRT-PCR reactions were carried out on a BioRad CFX96 qPCR System using the TB Green Premix Ex Taq (Takara). Four biological replications were carried out for each treatment, each with two technical replicates. The relative expression levels were normalized to actin and calculated using the log_2_(2^−ΔΔCT^). Supplementary Table S1 provides the gene-specific primer used for qRT-PCR.

### 2.11. Statistical Analysis

The experiment was performed in a completely randomized design with four replicates per treatment. Duncan’s multiple range test was used to compare the means of separate replicates and the statistical significance was established at *p* < 0.05. All statistical measures were performed using SAS 9.1.3 (SAS Institute Inc., Cary, NC, USA).

## 3. Results

### 3.1. Leaf Water Potential, H_2_O_2_ Concentration and Antioxidant Enzyme Activities

Drought rapidly decreased leaf water potential (*Ψw*) up to −1.34 MPa after 4 days of drought stress, whereas no significant difference occurred in the control plants (Table 1). Drought caused H_2_O_2_ to accumulate from 4 days of the experiment, showing a 2.5-fold increase at day 14, compared to the control. However, the H_2_O_2_ concentration remained unchanged in the control plants throughout the experimental period (Table 1). Leaf biomass started to decrease after 4 days of drought treatment and then significantly decreased by 42.6% of control at day 14. No significant difference was observed between treatments in root biomass (Table 1).

**Figure 1:**
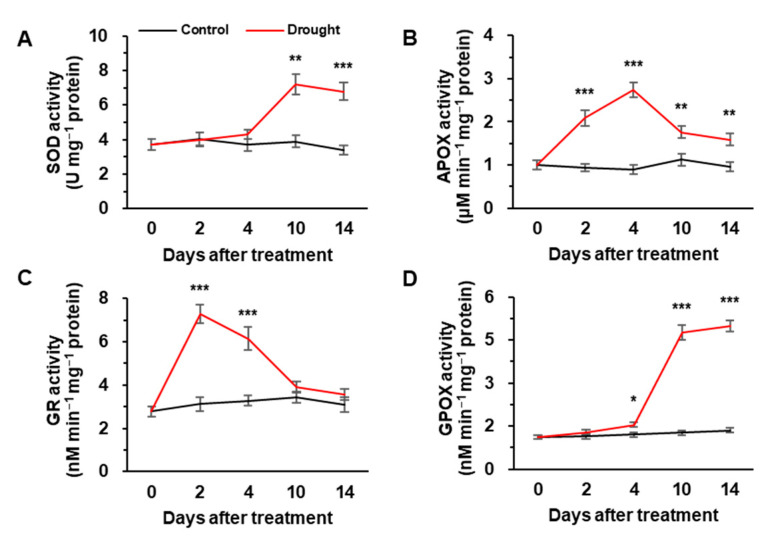
Changes in activities of superoxide dismutase (SOD, (**A**)), ascorbate peroxidase (APOX, (**B**)), glutathione reductase (GR, (**C**)) and glutathione peroxidase (GPOX, (**D**)) in the leaves of well-watered (control) or drought-stressed plants for 14 days. The black and red lines indicate control and drought stress, respectively. Data are represented as means ± S.E. for n = 4. Asterisks indicate a significant difference between well-watered and drought-stressed plants: * *p* < 0.05, ** *p* < 0.01, *** *p* < 0.001.

Under drought conditions, SOD and GPOX activity increased considerably to ˃1.9-fold from days 4–10 and was then maintained at a similar level until day 14 (Figure 1A,D). In contrast, APOX and GR activity was markedly increased up to 3.1-fold during the initial 4 days and 2 days of drought stress, respectively, and then largely decreased but remained slightly higher than the control values during the later stage of the drought (days 10–14) (Figure 1B,C). No significant changes in antioxidant enzyme activities were observed in the control plants.

### 3.2. ABA and Sulfate Concentrations in Leaves, Roots, and Xylem

**Figure 2:**
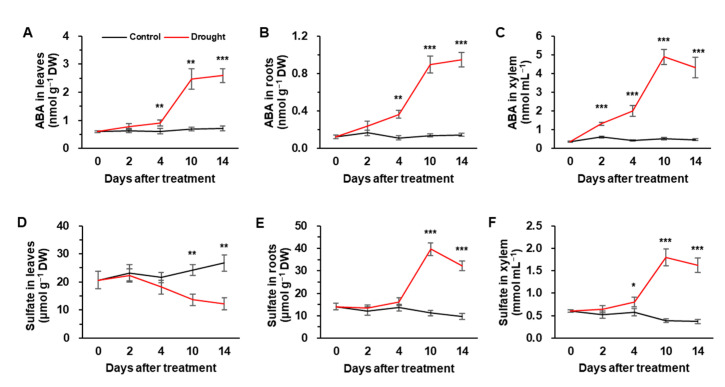
Changes in the concentration of ABA (**A**–**C**) and sulfate (**D**–**F**) in leaves (**A**,**D**), roots (**B**,**E**), and xylem (**C**,**F**) of well-watered (control) or drought-stressed plants for 14 days. The black and red lines indicate control and drought stress, respectively. Data represented as means ± S.E. for n = 4. Asterisks indicate a significant difference between well-watered and drought-stressed plants: * *p* < 0.05, ** *p* < 0.01, *** *p* < 0.001.

Endogenous ABA levels in leaves were significantly increased by drought stress, with a rapid increase of 3.6-fold at day 10 compared to the control (Figure 2A). Similarly, ABA concentrations in roots and xylem were gradually increased up to day 10 and were then maintained or slightly decreased in drought-stressed plants (Figure 2B,C). At day 14, the ABA concentration increased significantly in the phloem of drought-stressed plants (Supplementary Figure S1A). ABA concentration was not changed in the leaves, roots and xylem of control plants during the experimental period (Figure 2A–C). The sulfate concentration in the leaves of control plants markedly increased from days 4–14, whereas it showed the opposite trend in drought-stressed plants, resulting in a 50% of decrease compared to control (Figure 2D). Compared to the control plants, sulfate concentration in the roots and xylem of drought-stressed plants started to increase from day 4 and showed the largest increase (3.7 or 4.6-fold, respectively) at day 10 (Figure 2E,F). At day 14, sulfate concentration decreased markedly in the phloem of drought-stressed plants (Supplementary Figure S1B).

### 3.3. Cys and GSH Concentration, and the Expression of Sulfate Assimilation-Related Genes

**Figure 3:**
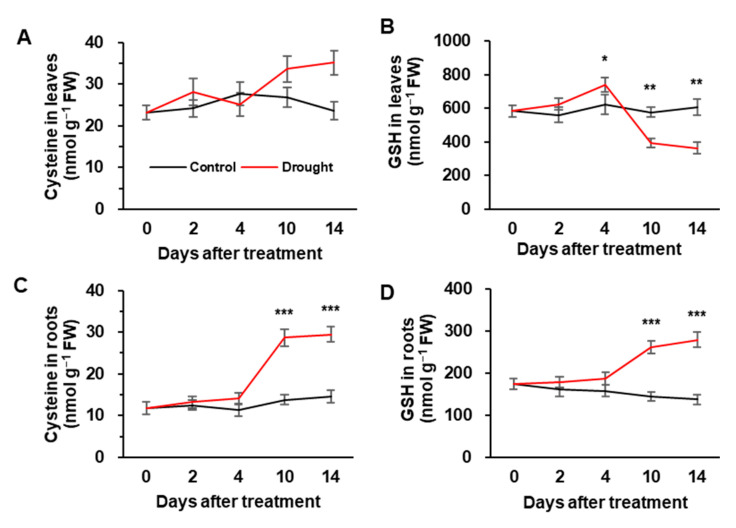
Changes in concentration of cysteine (**A**,**C**) and glutathione (**B**,**D**) in leaves (**A**,**B**) and roots (**C**,**D**) in well-watered (control) or drought-stressed plants for 14 days. The black and red lines indicate control and drought stress, respectively. Data are represented as means ± S.E. for n = 4. Asterisks indicate a significant difference between well-watered and drought-stressed plants: * *p* < 0.05, ** *p* < 0.01, *** *p* < 0.001.

**Figure 4:**
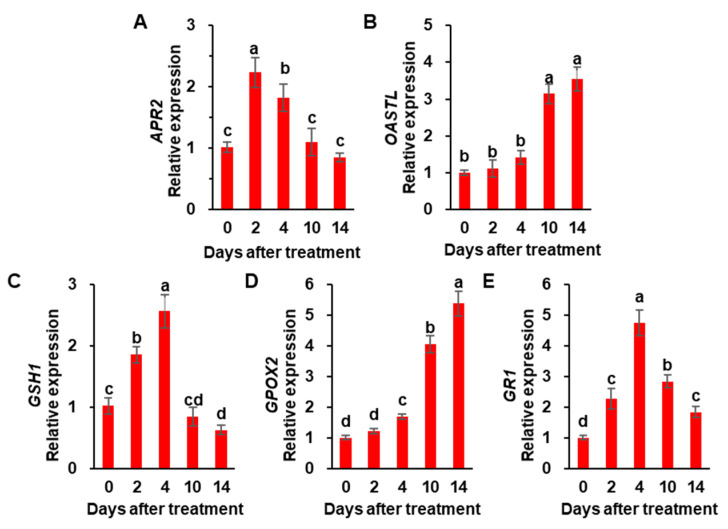
Relative expression of sulfate assimilation-related genes, adenosine-5-phosphosulfate reductase 2 (*APR2*, (**A**)), *O*-acetylserine(thiol)lyase (*OASTL*, (**B**)), γ-glutamylcysteine synthetase 1 (*GSH1*, (**C**)), glutathione peroxidase (*GPOX2*, (**D**)) and glutathione reductase (*GR1*, (**E**)) in leaves of drought-stressed plants for 14 days. The values in drought-stressed plants at day 0 were set to 1. Data are represented as means ± S.E. for n = 4. The different letters indicate values that ate significantly difference at *p* < 0.05 according to Duncan’s multiple range test.

There was no significant difference in Cys concentration between the control and drought-stressed plants during the early phase of the drought (days 0–4). Afterward, Cys concentration was greatly increased by 1.5- and 2-fold in leaves and roots of drought-stressed plants, respectively, compared to control plants (Figure 3A,C). GSH concentration in leaves gradually increased during the early phase of drought treatment and then significantly decreased by 37.7% of the initial level (Figure 3B). In roots, GSH concentration was largely increased after 4 days of drought stress and presented a 1.6-fold increase compared to the initial level (Figure 3D). No significant changes in GSH concentration were observed in the leaves and roots of control plants (Figure 3B,D). The expression of sulfate assimilation-related genes was observed in drought-stressed plants (Figure 4A). The expression of the adenosine-5-phosphosulfate reductase 2 (*APR2*) gene in drought-stressed plants was remarkably up-regulated to 2.2-fold in the first 2 days of drought stress, after which it continuously decreased to the control level (Figure 4A). Drought enhanced the expression of *O*-acetylserine(thiol)lyase (*OASTL*) and glutathione peroxidase (*GPOX2*) from day 4 onward and increased by 3.5- and 5.3-fold, respectively, at day 14 (Figure 4B,D). The expression of γ-glutamylcysteine synthetase 1 (*GSH1*) was rapidly up-regulated to 2.5-fold during the early phase of the drought, after which it decreased significantly to 60% of the initial level during the late stage (days 10–14) (Figure 4C). Similar to *GSH1*, the expression of glutathione reductase (*GR1)* was highly up-regulated only during the early stage (Figure 4E).

### 3.4. Redox Status under Drought Stress

The redox status in the leaves of control and drought-stressed plants is shown in Table 2 and the reduced GSH concentration remained the same, as shown in Figure 3B. Drought stress gradually increased GSSG concentration to 1.6- fold by day 14. The GSH/GSSG ratio increased slightly for the first 2 days of the drought treatment, after which it decreased significantly to <45.1% of the control during the late stage of the drought. During the 4 days of the early phase of the drought, NADPH concentration in the control plants remained unchanged, while it increased to 1.6-fold in drought-stressed plants; however, no significant difference was observed between control and drought-stressed plants during the late stage. NADP^+^ concentration in drought-stressed plants was highly increased to 1.6- and 1.9-fold at day 10 and day 14, respectively, compared to control plants. The ratio of NADPH/NADP^+^ increased up to day 4 and then rapidly decreased to 50% of the level in the control plants.

### 3.5. Correlation of Sulfate or ABA with Physiological Parameters Influenced by Drought Stress

The physiological relationship between sulfate or ABA, the identified *Ψ_w_* and H_2_O_2_ concentrations and their scavenging enzymes, sulfate assimilation-related gene expression, and redox statuses, as affected by drought treatment, are presented in Table 3. *Ψ_w_* was negatively correlated with sulfate and ABA concentration in the xylem, ABA, H_2_O_2_, GPOX, *OASTL*, and Cys, while being positively correlated with GR and redox status in the leaves of drought-stressed plants. Sulfate and ABA concentrations in the xylem were positively correlated with ABA, H_2_O_2_, GPOX, *OASTL*, and Cys, and were negatively correlated with sulfate, GR, *GSH1*, and redox status in leaves. Similar trends were observed in the correlation between ABA in leaves and other parameters, and the opposite trends were observed in correlation with sulfate in leaves.

## 4. Discussion

### 4.1. Drought Intensity-Responsive H_2_O_2_ Accumulation and Antioxidative Enzymatic Activity

The decrease in *Ψ_w_*, caused by hydraulic stresses such as drought and salinity, has proven to be responsible for the decrease in stomatal conductance and the consequent reduction in photosynthetic CO_2_ assimilation [30,31], mineral uptake and assimilation [10,25,32] and the cell extension process [26]. In the present study, drought imposition over 14 days by reducing daily irrigation volume, decreased *Ψ_w_* from −0.39 MPa to −1.34 MPa (Table 1). The *Ψ_w_* at day 14 is higher than the range of −2.27 MPa to −2.33 MPa measured in white clovers exposed to 4 weeks of drought [26] or −2.0 MPa measured in drought-susceptible common bean cultivars [33], and lower than the range of −0.78 MPa to −0.9 MPa recorded in eight *B. napus* cultivars exposed to 7 days of water-deficit treatment [25]. In a previous study, we found that drought treatment for 14 days successfully induced drought stress in *B. napus*, as evidenced by the minimum *Ψ_w_* value (−1.27 MPa), which resulted in the lowest photosynthetic activity accompanied with higher H_2_O_2_ accumulation and high lipid peroxidation levels [6]. In the present study, when the *Ψ_w_* was ˃−0.57 MPa during the early phase of drought (days 0–4), H_2_O_2_ accumulation was not considerably changed (Table 1) with a strong activation of APOX (Figure 1B) and GR (Figure 1C), indicating that the ascorbate-glutathione pathway was highly induced during the early phase of drought stress. It is well established that APOX plays an active role in scavenging H_2_O_2_, particularly under mild stress [26,34]. Enhancement of GR activity was prominent at the early stress period under drought stress [35] and pathogen-infected conditions [36]. Therefore, during the early phase of drought, the strong activation of APOX and GR might be an important facet of drought acclimation that permits the preservation of membrane integrity. During the late phase of drought stress (days 10–14; *Ψ_w_* ≤ −1.34 MPa), H_2_O_2_ accumulation was predominant (Table 1) and was accompanied with higher activation of SOD and GPOX activity (Figure 1C,F). Higher enhancement of SOD enzyme activity suggested that under oxidative stress, more H_2_O_2_ was needed to trigger the production of ROS by the electron transport chain; similar results were observed in *B. napus* exposed to 15 days of drought [27]. The activation of SOD at the late phase during the decrease in APOX and GR activity (Figure 1A–C) indicates that cell oxidation occurred. In addition, GPOX activity after 10 days of drought treatment (*Ψ_w_* ≤ −1.34 MPa) enhanced to approximately 4-fold compared to control plants (*Ψ_w_* ≥ −0.54 MPa) (Figure 1D). Similarly, overexpression of GPOX increased the rate of GSH generation and oxidation [37]. These results indicate that higher GPOX activation during the late drought phase is a factor for the cell-oxidizing process of reduced GSH to its disulfide form (GSSG) [38].

### 4.2. Drought Intensity-Responsive Changes in Sulfate, ABA Allocation, and S-Assimilates in Leaves

Drought generally decreases the mineral uptake kinetics per unit root, which might slow down nutrient uptake [39] or decrease the expression of nutrient-uptake proteins in roots [32]. In the present study, during the early 4 days of drought treatment, sulfate concentrations did not change markedly in either leaves or roots (Figure 2D,E), and the increase in GSH was significant only at day 4; however, during the late phase of drought stress (i.e., days 10–14; *Ψ_w_* ≤ −1.34 MPa), sulfate concentration in leaves decreased by approximately 50% (Figure 2D), accompanied with accumulation of Cys (Figure 3A) and a loss of GSH (Figure 3B) when compared to the control. A significant decrease in GSH in leaves during the late phase might be due to insufficient sulfate supply [15] and to the limiting role of glutathione synthetase [40]. Several points should be highlighted here: the abundance of sulfate in roots and in xylem sap (Figure 2E,F), and the Cys accumulation in both leaves and roots (Figure 3A,C) even though there was a significant decrease in the GSH pool in leaves, indicate that the availability of sulfate and/or Cys in leaves is not the exclusive determinant for the GSH system in leaves, which is possibly linked to other regulatory components. In fact, among the literature previously published, stress-responsive Cys and GSH concentrations have not been consistent, as the two major sulfate assimilates were affected by stress intensity, sulfur allocation into different organs and nutritional state [15,41,42].

It has been widely established that stress-induced ROS (especially H_2_O_2_) activates phytohormone signaling, and vice versa with their possible interactive regulatory functions [6,27,43,44]. Among these interactions, ABA-dependent signaling pathway has been studied in greater detail in different plants and stressors. For instance, H_2_O_2_-stimulated-ABA accumulation has shown to be a critical response to severe drought symptom development [6]. As expected, endogenous ABA levels began to increase in both leaves and roots from day 4 of drought stress, to a larger extent during the late phase (days 10–14; *Ψ_w_* ≤ −1.34 MPa) (Figure 2A,B), when H_2_O_2_ accumulation concurrently occurred (Table 1). Drought-induced H_2_O_2_ and ABA accumulation during the late phase was concomitant with a prominent increase in sulfate concentration in roots and in xylem sap but not in leaves (Figure 2D–F). Similarly, in Arabidopsis, sulfate application increased H_2_O_2_ levels in guard cells to a similar extent as the ABA level [20] by specific activation of plasma membrane-localized NADPH oxidases in an ABA-dependent manner [6,20]. In maize (*Zea mays* L.), sulfate was the sole ion that increased in xylem sap following a concomitant increase in xylem-borne ABA transport during 12 days of withholding water [18], leading to ABA-dependent stomatal closure [20,22]. Our previous studies have clearly shown that drought-responsive H_2_O_2_ accumulation with progressing drought stress intensity leads to an activation of ABA synthesis and its signaling [6,45,46]. Therefore, the enhanced ABA level is mainly due to H_2_O_2_-mediated activation of oxidative burst signaling (*OXI1*) and mitogen-activated protein kinases (*MAPKs*) [47], rather than sulfate alone (e.g., the decreased sulfate level in leaves). The enhanced ABA level and its signaling in tune activate H_2_O_2_ production leading to drought symptom development [6,45,46]. Consistent with the previous studies above mentioned, drought-enhanced ABA and sulfate in xylem during the late phase (Figure 2C,F) resulted in an increase in Cys (Figure 3A) and a decrease in GSH concentration in leaves (Figure 3B). These data strongly suggest that xylem-borne sulfate and/or ABA, which were enhanced especially during late stage of drought, might suppress the Cys incorporation into GSH leading to a loss of the GSH pool.

### 4.3. Sulfate and/or ABA Roles in Regulating Sulfate Assimilation

To further assess the regulatory roles of sulfate and/or ABA in the sulfate assimilatory pathway, the expression of genes involved in sulfate assimilation was measured in leaves exposed to drought. The expression of *APR2* was markedly enhanced only for the early 4 days, while suppressed during the late stage (days 10–14) in the leaves exposed to drought (Figure 4A). Consistently, in Arabidopsis, ABA did not affect the mRNA levels of *APR* isoforms and the regulation of APR was not changed in mutants deficient in ABA accumulation (*aba1* and *aba2*) or ABA signaling (*abi1* and *abi2*) [48]. It thus suggests that APR regulation during the late phase of drought is clearly ABA-independent, and that *APR2* has to be rapidly activated to allow Cys synthesis to meet the increased GSH level in the early drought phase (Figure 3A,B). In contrast, the *OASTL* expression was hardly activated in the early 4 days and then highly enhanced during the late stage of drought stress (Figure 4B). Barroso et al. [41] reported the salt-induced *OASTL* in an ABA-dependent manner and the external ABA-driven activation of *OASTL*. Therefore, *APR2* and *OASTL* act differently in regulating the drought-responsive sulfate assimilatory pathway: APR is involved in Cys synthesis as an acclamatory response in an ABA-independent manner, while *OASTL* is at the late stage of drought stress (Figure 4A,B). This study has shown a drought-responsive suppression of *GSH1* expression in the leaves at a late stage (days 10–14) (Figure 4C). Several results have shown that sulfate and/or Cys affect ABA synthesis and the endogenous level of ABA [18,19,20], and sulfate acts as a mobile signal stimulating Cys synthesis to trigger ABA synthesis and signaling [18,49]. The present data and those from past literature strongly suggest that during the late phase of drought-driven xylem, sulfate activates ABA synthesis and signaling in leaves and consequently leads to an ABA-mediated suppression of the GSH synthesis gene *GSH1* (Figure 4C), resulting in a decrease in GSH concentration.

### 4.4. Interactions between Sulfate and ABA in GSH-Based Redox Control

In the context of of an interactive function between sulfur and ABA in the stress-responsive biological action, especially in stomatal movement [18,19,20,49], we assessed the sulfate- and/or ABA-mediated GSH-based redox control. During the early phase of drought treatment (day 0–4), the resulting GSH/GSSG redox state transiently shifted toward a slightly reduced state on the initial 2 days. This transient increase in GSH (Figure 3B) and GSH/GSSG ratio at day 2 (Table 2) might be attributed to a strong activation of ROS scavenging enzymes in the ascorbate-glutathione cycle (APOX and GR; Figure 1B,C), as well as an enhanced expression of *GSH1* (Figure 4C), as an early acclimation reaction as shown by Tausz et al. [50]. During the late phase of drought stress (days 10–14, *Ψ_w_* ≤ −1.34 MPa), enzymatic activity of GR and expression of *GR1* were remarkably suppressed (Figure 1C and Figure 4E) in a concurrent increase in ABA (Figure 2A). Thus, the decreased GSH/GSSG ratio at this late phase (Table 2) would be attributed to ABA-mediated suppression of GR, which allows the recycling of GSSG back to GSH, using NADPH as the reducing power [51]. In contrast, GPOX activity and *GPOX2* gene expression were greatly increased during the late phase of drought stress (Figure 1D and Figure 4D). Furthermore, the activation of GPOX activity to drought intensity was nearly parallel to the increased ABA level in leaves and xylem sap (Figure 2A,C; Table 3), suggesting the ABA-mediated regulation of GPOX activity. In a genome-wide analysis of the GPOX gene family in *B. napus*, ABA-responsive enhancement of *BnGPOX1* under salinity and PEG-induced water stress conditions was characterized [52]. Therefore, during the late phase of drought (*Ψ_w_* ≤ −1.34 MPa), ABA-activated GPOX, with an antagonistic repression of GR, would be responsible for shifting GSH- and NADPH-based redox to more oxidative state along with enhanced H_2_O_2_ accumulation, consistent with a general concept of eco-physiological stress responses [50]. The present data clearly indicate that the loss of reducing potential in the GSH- and NADPH-based redox system, as a clear indicant of drought-induced oxidation process, might be attributed to the decrease in GSH synthesis and further oxidation of GSH catalyzed by GPOX with an antagonistic suppression of GR in a sulfate-activated ABA-dependent manner.

## 5. Conclusions

**Figure 5:**
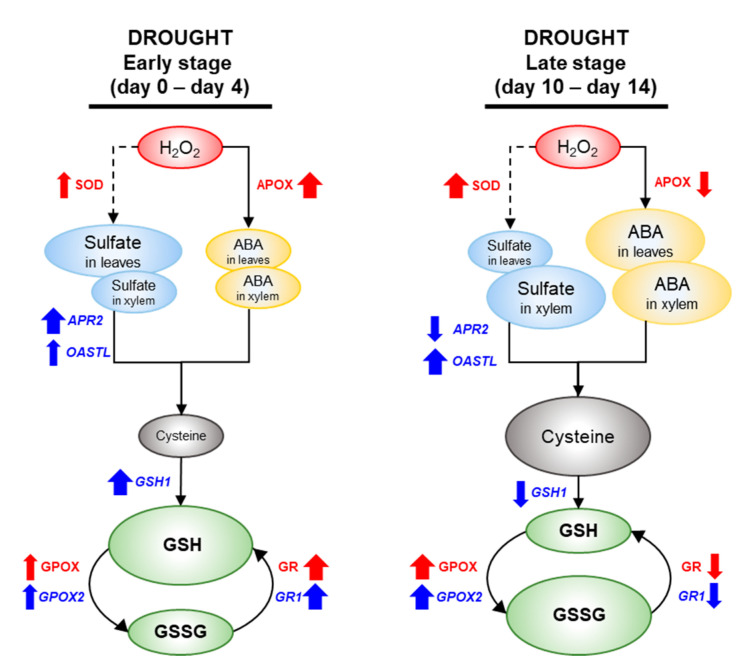
Schematic diagram for the proposed interactive regulation of sulfate to ABA-mediated regulation of sulfur assimilation and GSH redox in two distinct phase of drought intensity. Changes in metabolites, enzyme activities, and gene expressions in related to ROS scavenging, ABA, sulfur assimilation and GSH-based redox status are indicated by black, red, and blue letters, respectively. The circle size indicates the amount of metabolites. The thickness of the arrow indicates the strength of induced or depressed response.

A time-course analysis in relation to drought intensity clearly showed that drought-responsive changes in sulfate and ABA allocation in different organs would be an important stress response, which further coordinates the regulation of the sulfate assimilatory pathway toward GSH metabolism and the GSH redox control. Regression analysis revealed that the decrease in *Ψ_w_* with progression drought stress was closely correlated with the increase in sulfate and ABA level in xylem and leaves each other (Table 3). The physiological responses to drought intensity were distinguished by two distinct phases characterized by (1) a transient increase in GSH pool and in GSH/GSSG ratio with a strong activation of the H_2_O_2_ scavenging enzymes in the ascorbate-glutathione cycle (APOX and GR) for the early phase (days 0–4, *Ψ_w_* ≥ −0.54 MPa) and (2) a decrease in GSH with Cys accumulation resulting in a remarked loss of reducing potential in both GSH- and NADPH-based redox system, accompanied by a remarked GPOX activation with an antagonistic suppression of GR in a sulfate-activated ABA-dependent manner during the late phase (days 10–14; *Ψ_w_* ≤ −1.34 MPa) (Figure 5). To the best of our knowledge, the present findings are the first report of drought intensity-induced changes in sulfate and ABA coordinates in the regulation of sulfate assimilation toward GSH metabolism and GSH-based redox. Nevertheless, a critical point “sulfate-dependent *de novo* synthesis of ABA, as well as ABA-mediated redox control by ABA itself or by oxidative stress signal” needs to be well-defined.

## Figures and Tables

**Table 1:** Changes in the leaf water potential, H_2_O_2_ concentration, leaf and root biomass in the well-watered (control) or drought-stressed plants for 14 days.

Physiological Parameters	Days after Treatment				
/Treatment	0	2	4	10	14
Leaf water potential (*Ψ_w_*, MPa)					
Control	−0.39	−0.42	−0.45	−0.54	−0.53
Drought	−0.39	−0.49	−0.57 *	−1.12 **	−1.34 ***
H_2_O_2_ concentration (nmol g^−1^ FW)					
Control	2.97	3.06	3.16	3.08	3.47
Drought	2.97	3.54	3.85	7.86 ***	8.67 ***
Leaf biomass (Dry matter, g plant^−1^)					
Control	8.23	8.56	10.89	14.07	16.48
Drought	8.23	8.47	8.92 **	9.49 ***	9.46 ***
Root biomass (Dry matter, g plant^−1^)					
Control	5.86	5.09	6.28	7.14	8.16
Drought	5.86	5.35	6.05	6.64	7.23

Data represented as means ± S.E. for n = 4. Asterisks indicate significant difference between well-watered and drought-stressed plants at each harvest day: * *p* < 0.05, ** *p* < 0.01, *** *p* < 0.001.

**Table 2:** Changes in GSH- and NADPH-based redox status in the leaves of *Brassica napus* 14 days after drought imposition.

		GSH	GSSG	GSH/GSSG	NADPH	NADP^+^	NADPH/NADP^+^
Day	Treatment	(nmol g^−1^ FW)	(nmol g^−1^ FW)	Ratio	(nmol g^−1^ FW)	(nmol g^−1^ FW)	Ratio
0	Control	582 ± 35	44.3 ± 3.7	13.2 ± 0.7	3.2 ± 0.3	6.0 ± 0.4	0.53 ± 0.04
	Drought	582 ± 35	44.3 ± 3.7	13.2 ± 0.7	3.2 ± 0.3	6.0 ± 0.4	0.53 ± 0.04
2	Control	560 ± 46	45.5 ± 5.7	12.4 ± 0.6	3.7 ± 0.3	7.1 ± 0.6	0.53 ± 0.04
	Drought	697 ± 37 *	50.4 ± 4.3	13.9 ± 0.5 *	5.0 ± 0.4 *	6.9 ± 0.5	0.73 ± 0.09 *
4	Control	620 ± 58	48.1 ± 4.6	12.9 ± 0.8	3.3 ± 0.4	6.5 ± 0.4	0.51 ± 0.07
	Drought	741 ± 42 *	63.3 ± 5.2 *	11.7 ± 0.4	4.9 ± 0.3 **	7.3 ± 0.4	0.67 ± 0.07
10	Control	575 ± 30	51.2 ± 4.8	11.3 ± 0.5	3.6 ± 0.4	5.6 ± 0.5	0.65 ± 0.11
	Drought	392 ± 28 **	78.2 ± 5.9 **	5.1 ± 0.7 ***	3.0 ± 0.4	9.2 ± 0.8 **	0.33 ± 0.02 **
14	Control	606 ± 48	51.8 ± 4.9	11.7 ± 0.7	3.9 ± 0.3	5.4 ± 0.3	0.73 ± 0.08
	Drought	363 ± 33 **	81.4 ± 6.2 **	4.5 ± 0.6 ***	3.8 ± 0.3	10.3 ± 0.7 ***	0.37 ± 0.04 **

Data are represented as means ± S.E. for n = 4. Asterisks indicate significant difference between well-watered and drought-stressed plants at each harvest day: * *p* < 0.05, ** *p* < 0.01, *** *p* < 0.001.

**Table 3:** Linear correlations of sulfate and ABA with the descriptive parameters of H_2_O_2_ production and scavenging, sulfur assimilation and redox status as affected by drought stress for 14 days.

		Xylem		Leaves											
	*Ψ_w_*	Sulfate	ABA	Sulfate	ABA	H_2_O_2_	GR	GPOX	*OASTL*	*GSH1*	Cys	GSH	GSH/ GSSG	NADPH	NADPH/ NADP^+^
*Ψ_w_*	-	0.922 ***	−0.915 ***	0.866 ***	−0.972 ***	−0.965 ***	0.372	−0.966 ***	−0.974 ***	0.616 **	−0.830 ***	0.845 ***	0.958 ***	0.358	0.789 ***
Sulfate in xylem	-	-	0.963 ***	−0.847 ***	0.978 ***	0.959 ***	−0.405	0.975 ***	0.949 ***	−0.607 **	0.86 3 ***	−0.880 ***	−0.974 ***	−0.467 *	−0.826 ***
ABA in xylem	-	-	-	−0.821 ***	0.945 ***	0.929 ***	−0.234	0.955 ***	0.934 ***	−0.461 *	0.855 ***	−0.771 ***	−0.952 ***	−0.349	−0.765 ***
Sulfate in leaves	-	-	-	-	−0.837 ***	−0.874 ***	0.509 *	−0.857 ***	−0.898 ***	0.538 *	−0.694 ***	0.769 ***	0.864 ***	0.358	0.708 ***
ABA in leaves	-	-	-	-	-	0.971 ***	−0.389	0.983 ***	0.960 ***	−0.635 **	0.868 ***	−0.887 ***	−0.976 ***	−0.426	−0.816 ***

*Ψ_w_*, leaf water potential. The values measured in the drought stressed plants were used for correlation analysis. Correlation coefficient (r) and significant level of difference are reported; n = 15. * *p* < 0.05, ** *p* < 0.01, *** *p* < 0.001.

## Data Availability

Data is contained within the article and its Supplementary Materials.

## Supplementary Materials

The following supporting information can be downloaded at: https://www.mdpi.com/article/10.3390/metabo12121190/s1. Supplementary Table S1. Specific primers used for qRT-PCR. Supplementary Figure S1. Changes in concentration of ABA (A) and sulfate (B) in phloem of well-watered (control) or drought-stressed plant at day 14. Data represented as means ± S.E. for n = 4. Asterisks indicate a significant difference compared to the control (water application): * *p* < 0.05, *** *p* < 0.001.
