# Supplementary material for: Drought-Induced Xylem Sulfate Activates the ABA-Mediated Regulation of Sulfate Assimilation and Glutathione Redox in Brassica napus Leaves
Source: Metabolites. 2022 Nov 29;12(12):1190. doi: 10.3390/metabo12121190 (PMC9781433; doi:10.3390/metabo12121190)
Supplement: Supplementary file 1 [file metabolites-12-01190-s001.zip › metabolites-1999209-supplementary.pdf]

Supplementary Table S1. Specific primers used for qRT-PCR.

Target gene	GenBank Number	Forward sequence (5´-3´)	Reverse sequence (5´-3´)
BnAPR2	XM013836995	TACACGCAACGACTCTTTGG	GTCCTCAAGAAGTTCCAAAC
BnOASTL	AY096681	AAAACCCGGACCTCACAAGA	GAGTTTCCCGGCATTTTCAG
BnGSH1	AM265631	TGAATCAGGGGAAACAAAGC	TGACTTCAGCACAGGTTTGG
BnGPOX2	HM130682.1	TGAGTTTGAGCCAATTCAAAGG	GGAACTCAGCTTTGAACTTTGT
BnGR1	AF255651.1	CTGATTGGTGGGTTTTGCTT	ACCTTGGCACCATTGTTAGC
BnActin7	XM013867490	GATTCCGTTGCCCTGAAGTA	GCGACCACCTTGATCTTCAT

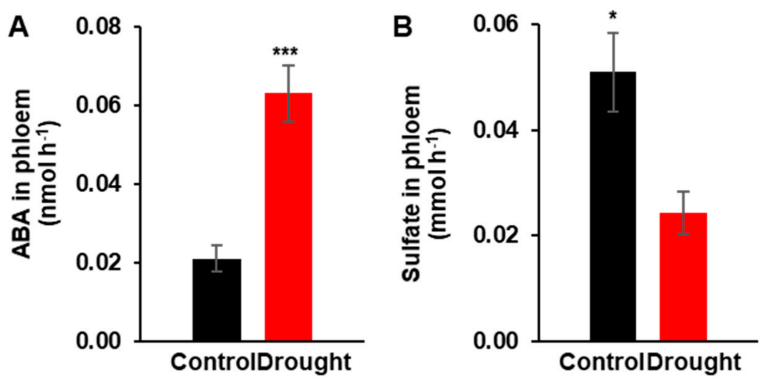

Supplementary Figure S1. Changes in concentration of ABA (A) and sulfate (B) in phloem of well-watered (control) or drought -stressed plant at day 14. Data represented as means ± S.E. for n = 4. Asterisks indicate a significant difference compared to the con trol (water application): * $p < 0.05$, *** $p < 0.001$.
